# Preprocedural Q-wave Depth Does Not Predict Mortality After Primary Coronary Intervention for ST-elevation Myocardial Infarction

**DOI:** 10.1016/j.cjco.2026.06.004

**Published:** 2026-06-18

**Authors:** Kristian Arak, Kaushika Rautray, Sulaiman Tomy, Rosemary Siby, Mhd Alaa Aldin Al Haffar, Salman Sahibzada, Julian P. Gunn, Hazel A. Haley

**Affiliations:** a Department of Cardiology, Northern General Hospital, Sheffield Teaching Hospitals NHS Foundation Trust, Sheffield, UK; b Division of Clinical Medicine, School of Medicine & Population Health, The University of Sheffield, Sheffield, UK

**Keywords:** angioplasty, depth, mortality, myocardial infarction, primary percutaneous coronary intervention, Q waves, ST-elevation myocardial infarction

## Abstract

**Background:**

Pathologic Q waves on the admission electrocardiogram (ECG) have historically been markers of myocardial necrosis and adverse outcomes after ST-elevation myocardial infarction (STEMI). Although quantitative markers such as Q-wave width may add prognostic value, they can be difficult to apply clinically. We hypothesised that maximal and average pre–percutaneous coronary intervention (PCI) Q-wave depth could predict short- and medium-term mortality.

**Methods:**

We performed a retrospective cohort study of consecutive STEMI patients undergoing primary PCI (N = 625). Pre-PCI ECGs were analysed using European Society of Cardiology/American Heart Association definitions to identify pathologic Q waves. Maximal and average Q-wave depth (mm) and infarct territory were recorded. Posterior patterns, bundle branch block confounding interpretation, and Q waves outside the culprit territory were excluded. Outcomes were all-cause mortality at 3 and 12 months.

**Results:**

Of the 625 patients studied, 203 with pathologic Q waves in the infarct territory had complete depth measurements for analysis. Twelve-month mortality was 7.4% (15 of 203). Neither maximal nor average Q-wave depth was associated with mortality at 3 or 12 months in any infarct territory. Pain-to-balloon time demonstrated a weak positive correlation with maximal (Spearman rho = 0.204, *P* = 0.004) and average (rho = 0.152, *P* = 0.033) Q-wave depth.

**Conclusions:**

In this small, observational cohort of STEMI patients undergoing primary PCI, preprocedural Q-wave depth was not associated with all-cause mortality at 3 or 12 months. These findings do not support Q-wave depth as a standalone pre-PCI prognostic marker; larger studies and alternative ECG measures may be more informative. Q-wave depth was weakly associated with total ischemic time, warranting further evaluation in larger multicentre cohorts.

## Résumé

**Contexte:**

Les ondes Q pathologiques observées sur l’électrocardiogramme (ECG) à l’admission ont historiquement été considérées comme des marqueurs de nécrose myocardique et de mauvais pronostic après un infarctus aigu du myocarde avec élévation du segment ST (STEMI). Bien que des marqueurs quantitatifs tels que la largeur de l’onde Q puissent apporter une valeur pronostique supplémentaire, leur application clinique peut s’avérer difficile. Nous avons émis l’hypothèse que la profondeur maximale et moyenne de l’onde Q avant l’intervention coronarienne percutanée (ICP) pourrait permettre de prédire la mortalité à court et moyen termes.

**Méthodologie:**

Nous avons mené une étude de cohorte rétrospective portant sur des patients consécutifs ayant subi un STEMI et traités par ICP primaire (N = 625). Les ECG pré-ICP ont été analysés à l’aide des définitions de la Société européenne de cardiologie et de l’American Heart Association afin d’identifier les ondes Q pathologiques. La profondeur maximale et moyenne des ondes Q (en mm) ainsi que le territoire de l’infarctus ont été enregistrés. Les profils postérieurs, les blocs de branche pouvant brouiller l’interprétation et les ondes Q situées en dehors du territoire coupable ont été exclus. Les critères d’évaluation étaient la mortalité toutes causes confondues à trois et douze mois.

**Résultats:**

Sur les 625 patients, 203 présentant des ondes Q pathologiques dans le territoire infarci disposaient de mesures complètes de la profondeur pour l’analyse. La mortalité à douze mois était de 7,4 % (15/203). Ni la profondeur maximale ni la profondeur moyenne des ondes Q n’étaient associées à la mortalité à trois ou douze mois, quel que soit le territoire de l’infarctus. Le délai entre l’apparition de la douleur et la mise en place du ballonnet a montré une faible corrélation positive avec la profondeur maximale (rho de Spearman = 0,204, *p* = 0,004) et moyenne (rho = 0,152, *p* = 0,033) de l’onde Q.

**Conclusions:**

Dans cette petite cohorte observationnelle de patients atteints d’un STEMI traités par ICP primaire, la profondeur de l’onde Q pré-interventionnelle n’était pas associée à la mortalité toutes causes confondues à 3 ou 12 mois. Ces résultats ne permettent pas de considérer la profondeur de l’onde Q comme un marqueur pronostique isolé avant l’ICP; des études de plus grande envergure et d’autres mesures ECG pourraient s’avérer plus informatives. La profondeur de l’onde Q était faiblement associée à la durée d’ischémie totale, ce qui justifie une évaluation plus approfondie dans des cohortes multicentriques plus larges.

The presence of pathologic Q waves on baseline electrocardiogram (ECG) has been demonstrated to be an independent predictor of adverse outcomes in ST-elevation myocardial infarction (STEMI) patients treated with percutaneous coronary intervention (PCI).^1^^,^^2^ In addition, they are superior to time from symptom onset in predicting these adverse outcomes and therefore provide additional value in staging the evolution of STEMI vs time from symptom onset or time to PCI alone.^1^^,^^2^ Beyond the mere presence of Q waves, their depth has been proposed as a more refined marker of myocardial injury, reflecting the severity and extent of necrosis. The Selvester score is a tool to estimate infarct size after myocardial infarction (MI), with higher scores correlating with increased left ventricular scarring,^3^ higher degree of left ventricular systolic dysfunction,^4^ and both poorer mortality and morbidity.^5^ However, in clinical practice, application of this scoring system may be time-consuming and therefore impractical. Other measures such as Q/R depth ratio across precordial leads have also been shown to add prognostic value in anterior STEMI, but this has not been demonstrated in other territories.^6^ In addition, Q-wave width does predict 90-day mortality and morbidity but only above certain millisecond thresholds, and longer term outcomes have not been fully explored.^7^

We therefore aimed to investigate the prognostic significance of pre-PCI Q-wave depth and territory in a contemporary STEMI cohort undergoing primary PCI at a high-volume tertiary centre. We hypothesised that greater Q-wave average and maximal depth would be associated with increased short- and medium-term mortality and offer an intuitive and simple way to calculate markers of adverse prognosis in STEMI patients that could be used in clinical decision-making.

## Methods

We conducted a retrospective observational cohort study of 625 patients diagnosed with STEMI and who underwent primary PCI at Sheffield Teaching Hospitals between January 2021 and August 2022. All patients treated within the predefined study period were included. The study size was therefore determined by the available cohort, and no formal prospective sample-size calculation was performed.

### Data collection

For each patient, demographic information, cardiovascular risk factors (ie, diabetes, hypertension, and smoking), and previous comorbidities (eg, previous MI, stroke, peripheral vascular disease, and chronic kidney disease) were documented, obtained from routinely collected clinical coding data. Data such as timing of PCI (eg, pain-to-balloon time), details of culprit lesion and date of death (if occurred) were also gathered from the institutional PCI database. Echocardiographic data were not collected for analysis due to inconsistent mode of scanning (bedside vs formal echocardiography) and lack of available reports for patients discharged to surrounding district hospitals.

### ECG analysis

For each patient, the pre-PCI ECG that was closest to time of balloon inflation was analysed by a single investigator who was blinded to clinical outcome. Pathologic Q waves were identified according to the following **E**uropean **S**ociety of **C**ardiology/**A**merican **H**eart **A**ssociation (ESC/AHA) definitions^8^: Any Q wave in leads V2-V3 > 0.02 second or QS complex in V2-V3 was considered pathologic, as were Q waves ≥ 0.03 and ≥ 1-mm deep or QS complexes in leads I, II, augmented vector left (aVL), augmented vector foot (aVF), or V4-V6, if present in 2 contiguous leads. For patients with pathologic Q waves, maximal Q-wave depth (mm), average Q-wave depth (mm), and anatomic territory data were recorded. Posterior MIs, ECGs with confounding bundle branch block, and Q waves outside the MI territory were excluded.

### Endpoints

The primary endpoint was all-cause mortality at 1 year. A secondary endpoint of all-cause mortality at 3 months was also assessed.

### Statistical analysis

Statistical analysis of the data was completed with IBM SPSS version 25 (IBM Corp, Armonk, NY). The final analysis cohort consisted of those patients with pathologic Q-wave in the STEMI territory and complete pre-PCI Q-wave depth measurements (N = 203). Continuous variables are presented as mean ± standard deviation and categorical variables are reported as number (%). Differences between those surviving 12 months and nonsurvivors were evaluated by independent-samples *t* test and Fisher exact test, respectively. Patients were stratified into tertiles according to the average and maximal Q-wave depths for the purpose of testing the main hypothesis. Mortality at 3 and 12 months was compared among these tertiles with Fisher exact test for the entire cohort and planned subgroup analysis of patients with anterior STEMI. Kaplan-Meier survival curves were used and the survival distributions were compared using the log-rank test when comparing time-to-event data. We developed 2 multivariable regression models to control for potential confounding variables on the association of Q-wave depth measured in the baseline 12-month mortality. The binary logistic regression model estimated an adjusted odds ratio (aOR), and the Cox proportional hazard regression model estimated an adjusted hazard ratio (aHR). Both models were adjusted based on the following variables: age, sex, diabetes, and hypertension. Other variables were excluded due to missing data or low event numbers, causing the regression models to become invalid. All analyses were considered statistically significant at a two-sided *P* value < 0.05. We undertook an additional analysis to assess the relationship between pain-to-balloon time and pre-PCI Q-wave depth (maximal and average) using the Spearman rank correlation in patients with available timing data (n = 194).

## Results

A total of 625 patients were studied. Of these, 102 did not have a pre-PCI ECG available or interpretable. Of the remaining 523 patients, 296 did not have pathologic Q waves or had confounding bundle branch blocks, and thus were excluded. Of those patients with pathologic Q waves, 24 were outside the STEMI territory, leaving 203 for inclusion in the analysis.

Of the 203 patients in the final cohort, 15 (7.4%) died within 12 months. As shown in Table 1, older age was the only baseline variable significantly associated with 12-month mortality (mean 70.3 vs 62.1 years; *P* = 0.007). In addition, neither average (*P* = 0.660) nor maximal (*P* = 0.908) pre-PCI Q-wave depth was significantly different between those who survived and those that did not. In addition, there were no significant differences in sex, comorbidities, or smoking status.

**Table 1:** Baseline characteristics of the final patient cohort (N = 203)

Characteristic	Survived (N = 188)	Died (N = 15)	*P* value
Age (years), mean ± SD	62.1 ± 11.3	70.3 ± 11.6	0.007∗
Average Q-wave depth (mm), mean ± SD	6.0 ± 4.6	6.5 ± 4.7	0.660
Maximal Q-wave depth (mm), mean ± SD	7.5 ± 4.9	7.6 ± 5.0	0.908
Male sex, n (%)	143 (76.1%)	13 (86.7%)	0.528†
**Comorbidities, n (%)**			
Diabetes mellitus	42 (22.3%)	6 (40.0%)	0.126†
Hypertension	82 (43.6%)	9 (60.0%)	0.283†
Chronic kidney disease	10 (5.3%)	2 (13.3%)	0.219†
Previous MI	18 (9.6%)	3 (20.0%)	0.192†
Multivessel disease‡	22 (12.2%)	4 (28.6%)	0.098†
**Smoking status, n (%)**§			0.263†
Never smoked	45 (36.3%)	3 (37.5%)	
Ex-smoker	30 (24.2%)	0 (0.0%)	
Current smoker	50 (40.3%)	5 (62.5%)	

*P* values were derived from independent-samples *t* test for continuous variables and Fisher exact test for categorical variables. The χ^2^ test was not used for any continuous variables used due to the low event rate.

MI, myocardial infarction; SD, standard deviation.

∗ Statistically significant (*P* < 0.05).

† Fisher exact test used.

‡ Analysis based on 195 patients with available angiographic data.

§ Analysis based on 133 patients with known smoking status (125 survived, 8 died).

Table 2 shows that both average and maximal Q-wave depth did not have any association with 3- or 12-month mortality. For the average Q-wave depth, the highest tertile did have the highest mortality at both 3 and 12 months, but this was not statistically significant (*P* = 0.332 and *P* = 0.475, respectively). The maximal Q-wave depth showed no correlation with 3-month mortality (*P* = 0.736) or 12-month mortality (*P* = 0.944).

**Table 2:** Mortality rates by tertiles of pre-PCI Q-wave depth (all territories)

Q-wave depth tertile	N	3-Month mortality, n (%)	*P* value∗	N	12-Month mortality, n (%)	*P* value∗
Average depth			0.192			0.475
Tertile 1 (lowest)	70	4 (5.7%)		70	5 (7.1%)	
Tertile 2 (middle)	65	1 (1.5%)		65	3 (4.6%)	
Tertile 3 (highest)	68	6 (8.8%)		68	7 (10.3%)	
Maximal depth			0.736			0.944
Tertile 1 (lowest)	67	5 (7.5%)		67	5 (7.5%)	
Tertile 2 (middle)	72	3 (4.2%)		72	6 (8.3%)	
Tertile 3 (highest)	64	3 (4.7%)		64	4 (6.3%)	

PCI, percutaneous coronary intervention.

∗ Fisher exact test for the association between Q-wave tertiles and mortality.

Table 3 provides a summary of a subgroup analysis of 108 patients with anterior STEMI. In this subgroup, the highest average Q-wave tertiles showed a trend toward higher 12-month mortality (11.1% in the highest vs 2.9% in the lowest), as did the maximal Q-wave tertiles (10.3% in the highest vs 2.9% in the lowest), but this finding was not statistically significant (*P* = 0.479). All other analyses of both average and maximal Q-wave depth in the remaining territories also showed no statistically significant associations with mortality at 3 or 12 months.

**Table 3:** Mortality rates by tertiles of pre-PCI Q-wave depth (anterior STEMI only, N = 108)

Q-wave depth tertile	N	3-Month mortality, n (%)	*P* value∗	N	12-Month mortality, n (%)	*P* value∗
Average depth			0.697†			0.479†
Tertile 1 (lowest)	34	1 (2.9%)		34	1 (2.9%)	
Tertile 2 (middle)	38	3 (7.9%)		38	3 (7.9%)	
Tertile 3 (highest)	36	3 (8.3%)		36	4 (11.1%)	
Maximal depth			0.625†			0.480†
Tertile 1 (lowest)	35	1 (2.9%)		35	1 (2.9%)	
Tertile 2 (middle)	34	3 (8.8%)		34	3 (8.8%)	
Tertile 3 (highest)	39	3 (7.7%)		39	4 (10.3%)	

PCI, percutaneous coronary intervention; STEMI, ST-elevation in myocardial infarction.

∗ Fisher exact test used for association between Q-wave tertiles and mortality within the anterior STEMI subgroup.

† Statistically significant (*P* < 0.05).

A multivariable logistic regression analysis was performed to assess for independent predictors and is presented in Table 4. By adjusting for age, sex, diabetes, and hypertension, there were no significant associations between average Q-wave depth (aOR = 1.008, 95% 0.894-1.136, *P* = 0.902) or maximal Q-wave depth (aOR = 0.988, 95% confidence interval 0.885-1.104, *P* = 0.836) and 12-month mortality. However, in both models, age was found to be a statistically significant independent predictor (aOR for average depth = 1.074, *P* < 0.05; aOR for maximal depth = 1.076, *P* < 0.05).

**Table 4:** Multivariable logistic regression analysis for predictors of 12-month mortality

Variable	aOR	95% CI	*P* value
Model 1: Average Q-wave depth (N = 203)∗			
Average Q-wave depth (per mm)	1.008	0.894-1.136	0.902
Age (per year)	1.074	1.016-1.135	0.011†
Model 2: Maximal Q-wave depth (N = 203)∗			
Maximal Q-wave depth (per mm)	0.988	0.885-1.104	0.836
Age (per year)	1.076	1.018-1.137	0.010†

aOR, adjusted odds ratio; CI, confidence interval.

∗ Models were adjusted for age, sex, diabetes, and hypertension.

† Statistically significant (*P* < 0.05).

The time-to-event analyses are presented. As shown in Figure 1, Kaplan-Meier curves did not show any difference in survival based on tertiles of average Q-wave depth (log-rank *P* = 0.451). In addition, a multivariable Cox proportional hazard regression model (Table 5) indicated that average and maximal Q-wave depth were not independent predictors of time-to-event mortality (aHR for average depth = 1.005, *P* = 0.937; aHR for maximal depth = 0.986, *P* = 0.796). Age continued to be the only significant independent predictor of mortality risk in both models (aHR = 1.070 and aHR = 1.072, respectively; *P* < 0.05).

**Figure 1:**
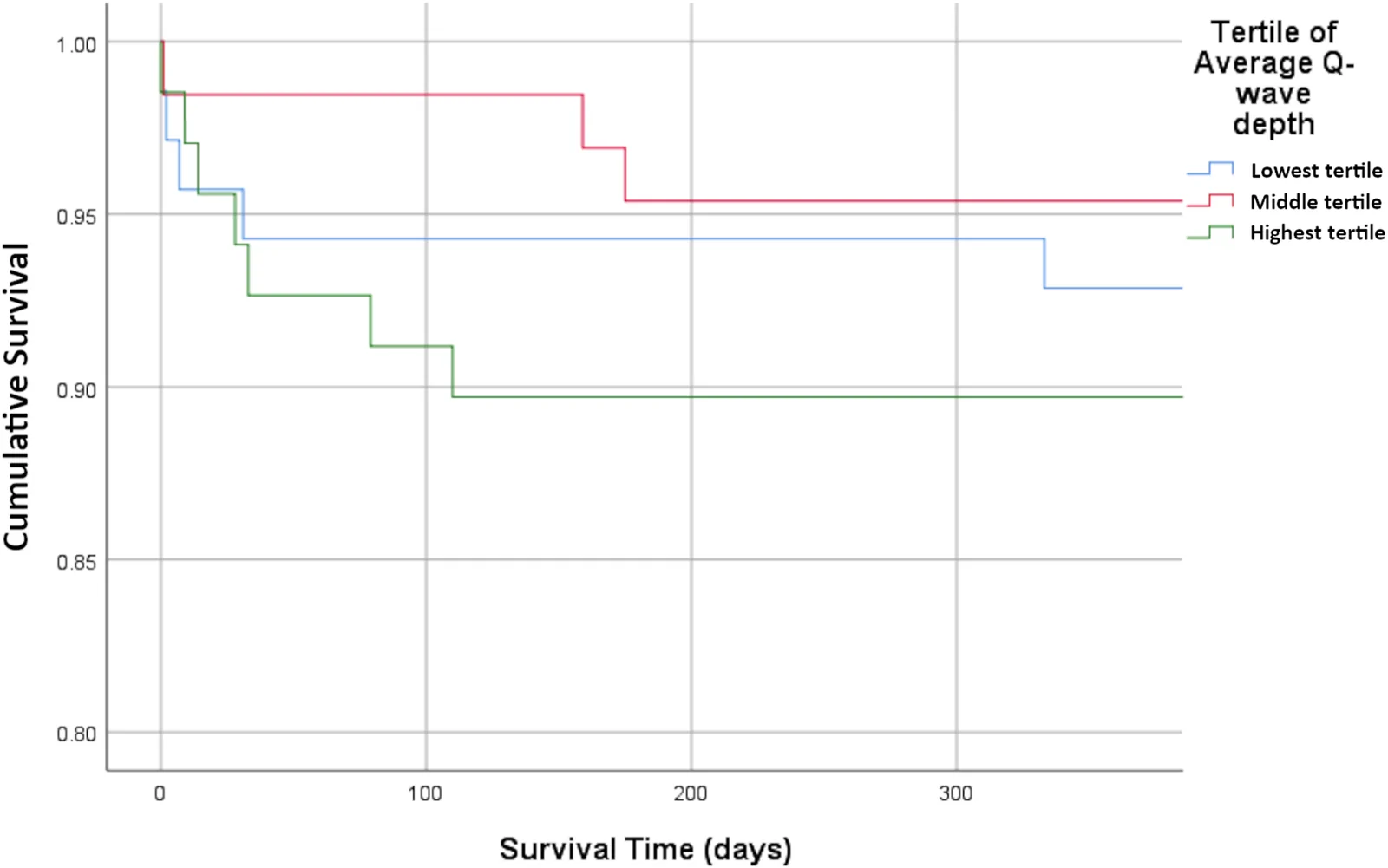
Kaplan-Meier survival curves for 12-month all-cause mortality, stratified by tertiles of pre–percutaneous coronary intervention average Q-wave depth. Average Q-wave depths: 2 mm (**blue**), 5 mm (**red**), and 11.2 mm (**green**).

**Table 5:** Multivariable Cox regression analysis for predictors of 12-month mortality

Variable	aHR	95% CI	*P* value
Model 1 (N = 203)∗			
Average Q-wave depth (per mm)	1.005	0.897-1.125	0.937
Age (per year)	1.070	1.016-1.127	0.010†
Model 2 (N = 203)∗			
Maximal Q-wave depth (per mm)	0.986	0.888-1.095	0.796
Age (per year)	1.072	1.018-1.130	0.009†

aOR, adjusted odds ratio; CI, confidence interval.

∗ Models adjusted for age, sex, diabetes, and hypertension.

† Statistically significant (*P* < 0.05).

### Pain-to-balloon time and Q-wave depth

Pain-to-balloon time revealed a statistically significant but weak positive correlation with both maximal Q-wave depth (Spearman rho = 0.204, *P* = 0.004) and average Q-wave depth (rho = 0.152, *P* = 0.033), indicating that longer ischemic time was weakly associated with deeper Q waves (Table 6; Figures 2 and 3).

**Figure 2:**
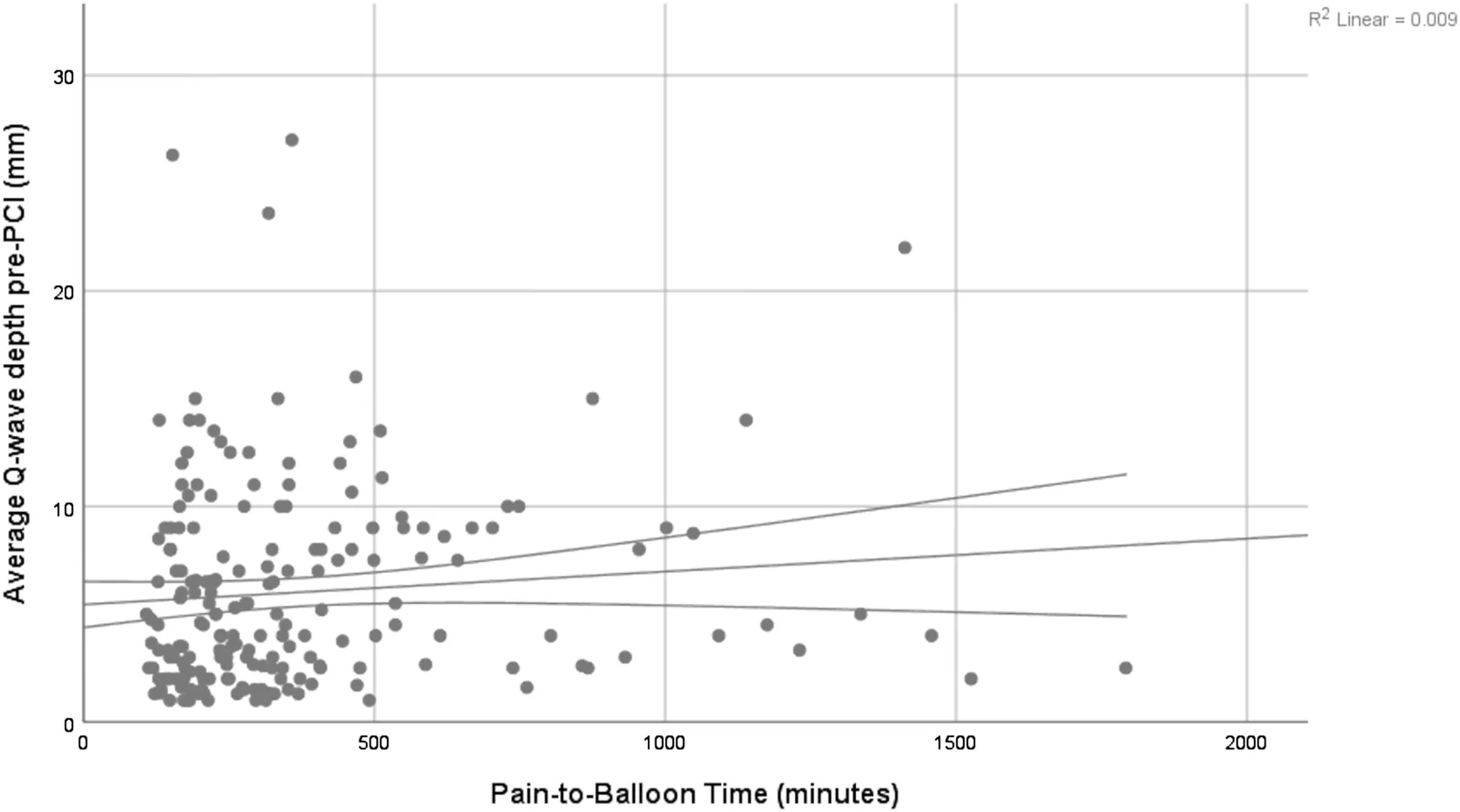
Average Q-wave depth by pain-to-balloon time. PCI, percutaneous coronary intervention.

**Figure 3:**
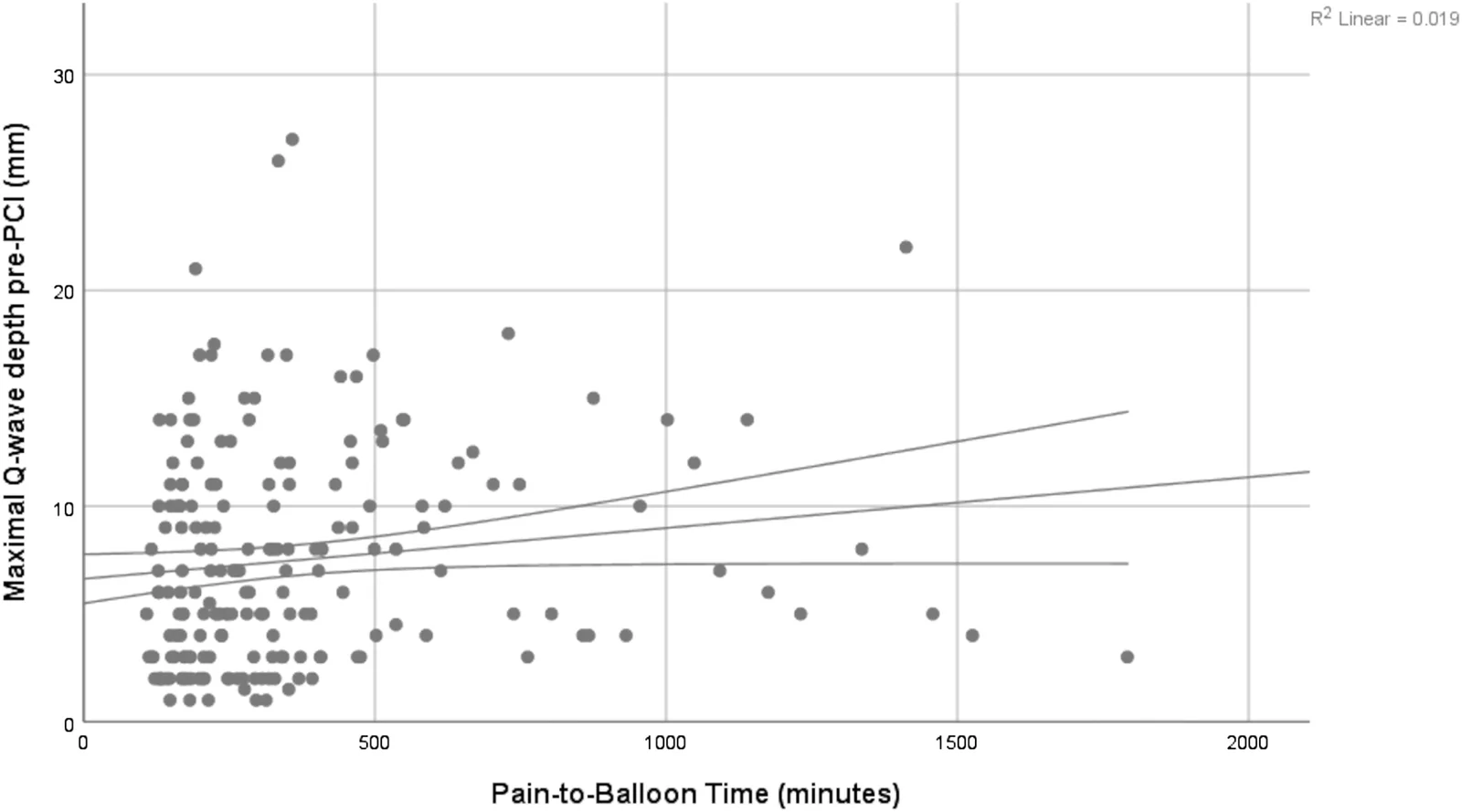
Maximal Q-wave depth by pain-to-balloon time. PCI, percutaneous coronary intervention.

**Table 6:** Correlation between pain-to-balloon time and Q-wave depth (N = 194)

Variable pair	Correlation coefficient (Spearman rho)	*P* value
Pain-to-balloon time vs maximal Q-wave depth	0.204	0.004∗
Pain-to-balloon time vs average Q-wave depth	0.153	0.033∗

∗ Statistically significant (*P* < 0.05).

As shown in Table 7, patients who died during follow-up demonstrated longer mean pain-to-balloon times compared with survivors (375.0 ± 262.4 vs 319.5 ± 232.5 minutes), although this difference did not reach statistical significance (*P* = 0.174).

**Table 7:** Comparison of ischemic time (pain-to-balloon) by mortality status

Variable	Survived (N = 568)	Died (N = 35)	*P* value∗
Pain-to-balloon time (min), mean ± SD	319.5 ± 232.5	375.0 ± 262.4	0.174

SD, standard deviation.

∗ Independent-samples *t* test.

Analysis of Q-wave evolution after PCI demonstrated that baseline Q waves resolved in a minority of patients after reperfusion therapy, as shown in Table 8. There was no statistically significant association between Q-wave dynamic status and 12-month mortality (*P* = 0.277), although interpretation is limited by the small sample size and low event rate within individual subgroups.

**Table 8:** Q-wave dynamics (pre- to post-PCI) and 12-month mortality

Q-wave dynamic status	Total number	Deaths, n (%)	*P* value∗
Resolved (pre-PCI yes, post-PCI no)	20	3 (15.0%)	0.277
Persisted (pre-PCI yes, post-PCI yes)	177	10 (5.6%)	
New-onset post-PCI (pre-PCI no, post-PCI yes)	139	6 (4.3%)	
No Q waves (pre-PCI no, post-PCI no)	250	16 (6.4%)	

PCI, percutaneous coronary intervention.

∗ Fisher exact test used across all categories.

### Characteristics of patients who died

Among the 15 patients who died during follow-up, early mortality clustered in those with severe presenting physiology. Four patients died within 10 days of presentation in the context of cardiac arrest, cardiogenic shock, and/or high-grade conduction disturbance. Of the remaining 11 deaths, 5 patients experienced prolonged ischemic time (delayed presentation and/or prolonged call-to-balloon time). The remaining 6 deaths did not share a consistent pattern of baseline, presentation, or procedural factors on descriptive review. Illustrative pre-PCI ECG examples are shown in Figure 4, comparing markedly deep Q waves in a patient who survived beyond 12 months with small Q waves in a patient who died during follow-up.

**Figure 4:**
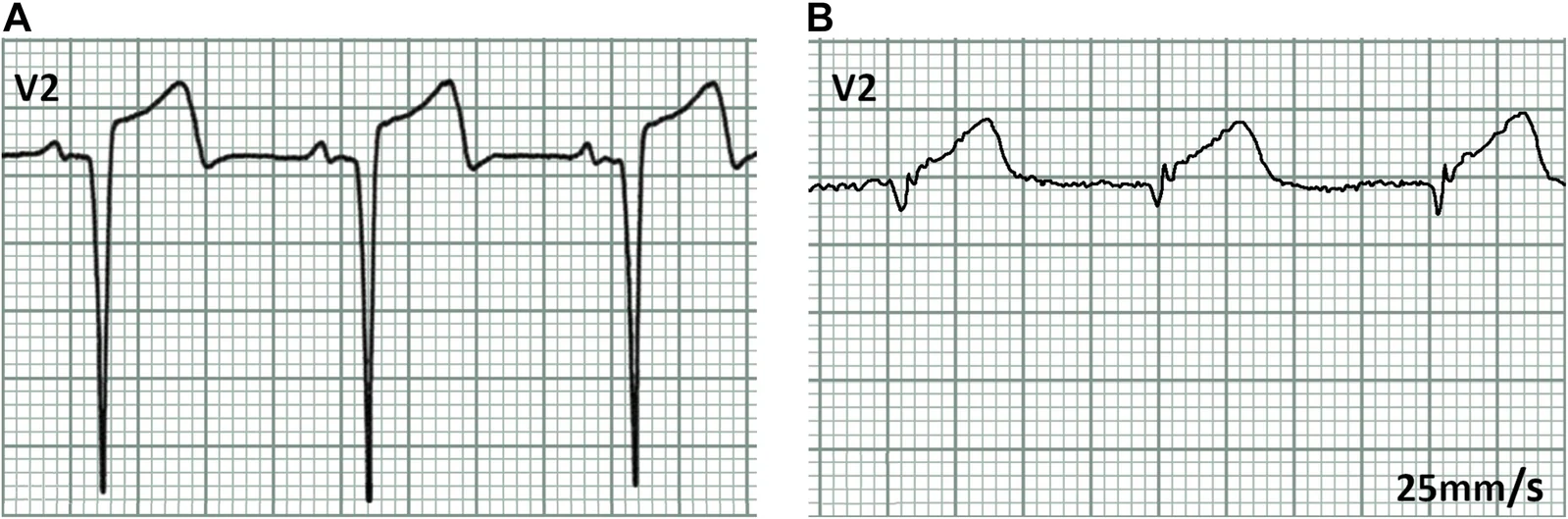
Illustrative pre–percutaneous coronary intervention electrocardiographic examples. Q waves 26 mm deep in a patient (A) who survived beyond 12 months vs Q waves 2 mm deep in a patient (B) who died during follow-up. These are illustrative examples and outcomes are not implied by Q-wave depth.

## Discussion

In this contemporary cohort of patients with STEMI undergoing primary PCI, neither maximal nor average pre-PCI Q-wave depth was associated with all-cause mortality at 3 or 12 months. Although pathologic Q waves have long been regarded as markers of established myocardial necrosis and adverse outcomes, our findings suggest that Q-wave depth alone does not confer meaningful prognostic value in the PCI era.

Prior studies have demonstrated that the presence of baseline Q waves predicts larger infarct size, reduced left ventricular function, and increased mortality.^1^, ^2^, ^3^, ^4^, ^5^ More refined ECG metrics---namely Q-wave width, Q/R ratios, and the Selvester QRS score---have shown stronger associations with infarct size and post-MI outcomes, but are more cumbersome to calculate.^3^, ^4^, ^5^, ^6^, ^7^ These parameters may reflect more complex conduction abnormalities and infarct transmurality than depth alone. The lack of association in our study suggests that Q-wave depth may capture only a limited proportion of the pathophysiologic substrate contributing to clinical risk. Depth may also be less robust as it depends on lead placement, baseline voltage, and body habitus.

Furthermore, although pathologic Q waves have traditionally been regarded as persistent markers of irreversible myocardial necrosis, contemporary reperfusion therapy has demonstrated that Q-wave evolution may be dynamic, with partial or complete resolution occurring after successful reperfusion. This phenomenon was also observed in our cohort, in which a minority of patients demonstrated resolution of Q waves after PCI. Alongside other factors, such as shorter total ischemic times and optimised secondary prevention strategies, modern reperfusion therapy may attenuate the prognostic significance of ECG markers that historically reflected irreversible myocardial damage. This may partially explain the lower-than-expected mortality and the weaker relationship between Q-wave characteristics and outcomes in this cohort.

Our additional analysis demonstrated pain-to-balloon time was associated with deeper Q waves, although the relationship was weak, suggesting Q-wave depth captures only a small component of ischemic duration and is unlikely to be clinically useful as a standalone marker. Although patients who died during follow-up demonstrated numerically longer pain-to-balloon times, this association did not reach statistical significance, likely reflecting the relatively low event rate and limited statistical power of this cohort.

### Territory-specific observations

In anterior STEMI, we observed a nonsignificant trend toward higher mortality with increasing Q-wave depth. Given the larger myocardial mass at risk and the established relationship between anterior infarct size and outcomes, a biologic signal in this subgroup is plausible. However, the absence of statistical significance and the lack of any trend in inferior or lateral STEMI indicate that territory-specific prognostic implications remain uncertain and require larger data sets for confirmation.

### Study limitations

The primary limitation of this study is the low event rate of only 15 deaths, which reduces the power to detect modest associations and increases the likelihood of type II error in this relatively small cohort, and our findings would benefit from validation in a larger STEMI cohort with higher event rate. Nevertheless, the number is large enough to show that, on an individual patient basis, Q-wave depth alone is not a reliable marker of prognosis. The number of available pre-PCI ECGs was also limited, with missing ECGs having the potential to be nonrandom (eg, patients who suffered cardiac arrest). In addition, we did not evaluate associations with infarct size, left ventricular systolic dysfunction, or major adverse cardiovascular events, which may be more sensitive markers of adverse myocardial remodelling than mortality alone. Finally, because analyses were restricted to patients with pathologic Q waves in the culprit territory, our findings may not be generalisable to all STEMI patients.

## Conclusions

In a contemporary PCI-treated STEMI population, in patients with pathologic Q waves at presentation, maximal and average pre-PCI Q-wave depths were not associated with short- or medium-term mortality. Our findings do not support using the depth of Q waves on the presenting ECG to triage patients away from primary PCI, and it should not be used in isolation to delay or withhold reperfusion therapy. More comprehensive ECG-derived indices of myocardial injury, particularly Q-wave width and multiparametric QRS scoring systems, may have greater clinical utility and should be the focus of future prognostic research. Q-wave depth was associated with total ischemic time, yet this relationship was weak. Given low event rate of only 15 deaths within this smaller cohort, our results would benefit from being validated in larger, multicentre studies.

## Rights Retention Statement

For the purpose of open access, the author has applied a Creative Commons Attribution (CC BY) licence to any Author Accepted Manuscript version arising from this submission.

## Data Availability

The data underlying this study are not publicly available due to patient confidentiality. De-identified data may be made available from the corresponding author on reasonable request.

## Ethics Statement

This paper has adhered to the Strengthening the Reporting of Observational Studies in Epidemiology (STROBE) guidelines for cohort studies.

## Patient Consent

The authors confirm that patient consent is not applicable to this article. This retrospective cohort study used routinely collected, de-identified clinical data. No identifiable patient information is included in this work. Formal written consent for publication was not obtained because no identifiable individual patient details are reported.
